# Cortical–Hypothalamic Integration of Autonomic and Endocrine Stress Responses

**DOI:** 10.3389/fphys.2022.820398

**Published:** 2022-02-11

**Authors:** Derek Schaeuble, Brent Myers

**Affiliations:** Department of Biomedical Sciences, Colorado State University, Fort Collins, CO, United States

**Keywords:** hypothalamic–pituitary–adrenal axis, sympathetic, cardiovascular, medial prefrontal cortex, insula

## Abstract

The prevalence and severity of cardiovascular disease (CVD) are exacerbated by chronic stress exposure. While stress-induced sympathetic activity and elevated glucocorticoid secretion impair cardiovascular health, the mechanisms by which stress-responsive brain regions integrate autonomic and endocrine stress responses remain unclear. This review covers emerging literature on how specific cortical and hypothalamic nuclei regulate cardiovascular and neuroendocrine stress responses. We will also discuss the current understanding of the cellular and circuit mechanisms mediating physiological stress responses. Altogether, the reviewed literature highlights the current state of stress integration research, as well unanswered questions about the brain basis of CVD risk.

## Introduction

Cardiovascular disease (CVD) is the leading cause of death worldwide. Although diet, exercise, and other lifestyle risk factors have been extensively characterized, less focus has been placed on the effects of prolonged mental stress to increase CVD morbidity and mortality (Barefoot et al., 1996; Chida and Steptoe, 2010). Notably, CVD risk more than doubles with chronic stress exposure (Yusuf et al., 2004; Steptoe and Kivimäki, 2012); yet, the neurobiological mechanisms linking stress and cardiovascular health outcomes are only partially understood. However, recent methodological advances have improved the ability to specifically interrogate stress-responsive neurocircuitry and subsequent regulation of cardiovascular physiology.

The physiological stress response is a conserved biological mechanism that promotes survival and adaptation in the presence of potential threats to homeostasis. In response to stressors, the brain activates both autonomic and endocrine output to mobilize energy resources (Myers et al., 2014b). The immediate response is generated by the autonomic nervous system, composed of the sympathetic and parasympathetic branches. The sympathetic nervous system is governed by descending cortical and forebrain circuits that regulate the activity of pre-sympathetic neurons in the hypothalamus and brainstem which communicate with spinal pre-ganglionic sympathetic neurons (Ulrich-Lai and Herman, 2009). Post-ganglionic sympathetic neurons then promote fight-or-flight responses including elevations of epinephrine, glucose, heart rate, and blood pressure. The parasympathetic branch is regulated by descending cortical and forebrain innervation of brainstem pre-ganglionic nuclei which typically withdraw activity in response to stress. On a prolonged timescale, the neuroendocrine hypothalamic–pituitary–adrenocortical (HPA) axis is activated by paraventricular hypothalamic corticotropin-releasing hormone (CRH; Herman et al., 2016). CRH is secreted in the anterior pituitary gland and stimulates the release of adrenocorticotropic hormone (ACTH). Systemic ACTH then acts on the adrenal cortex to stimulate synthesis and release of glucocorticoids. Glucocorticoids (cortisol in humans and corticosterone in rodents) act throughout the body *via* glucocorticoid receptors (GR) and mineralocorticoid receptors (MR; de Kloet et al., 2005). Importantly, corticolimbic circuits appraise context and prior experience to regulate autonomic activity, hormone secretion, and feedback through GR and MR signaling (Myers et al., 2012).

Although autonomic and endocrine stress responses are necessary for survival, exposure to traumatic or chronic stress can lead to autonomic imbalance, impaired negative feedback of the HPA axis, and illness (McEwen and Stellar, 1993; Herman et al., 2003). While the autonomic nervous system and HPA axis act through different mechanisms, central integration of both systems is necessary for appropriate physiological control. There is a limited collection of forebrain and brainstem structures that provide monosynaptic input to both autonomic pre-ganglionic neurons and HPA axis neurosecretory cells (Ulrich-Lai and Herman, 2009). In addition to the paraventricular hypothalamus (PVN), which houses both pre-autonomic and secretory cells (Swanson and Sawchenko, 1983), subsets of neurons in the bed nuclei of the stria terminalis, hypothalamus, and hindbrain (ventrolateral medulla and nucleus of the solitary tract) regulate neuroendocrine and autonomic outflow. Importantly, this stress regulation is modulated by descending corticolimbic inputs including the prefrontal and insular cortices, amygdala, and hippocampus (Herman et al., 2003; Myers, 2017). The hierarchy of cortical inputs to subcortical regions that innervate stress-effector cells is fundamental to translating cognitive appraisal and emotional processes into physiological activity. This review specifically focuses on stress-regulatory cortical regions and their targets in the hypothalamus. The amygdala, a temporal lobe limbic structure critical for the expression of fear, also provides multi-synaptic excitatory drive to both sympathetic and HPA axis stress responses (Swanson and Petrovich, 1998; Sah et al., 2003). The complex nuclear heterogeneity of the amygdala gives rise to numerous projections that target the bed nuclei of the stria terminalis, hypothalamus, and brainstem (Hermann et al., 1997; Prewitt and Herman, 1998; Saha, 2005; Myers et al., 2014a) Although, the actions of specific amygdaloid nuclei on autonomic and endocrine stress responses have been extensively reviewed elsewhere (Davis, 1997; Shekhar et al., 2003; Phelps and LeDoux, 2005; Ulrich-Lai and Herman, 2009; Davis et al., 2010; Myers et al., 2012, 2014b; Herman et al., 2016; Myers, 2017). Accordingly, the following review will concentrate on the rodent cortical–hypothalamic neurocircuitry that integrates neuroendocrine and cardiovascular autonomic activity. While the brainstem has a pivotal role in stress integration, this topic has also been reviewed elsewhere (Ulrich-Lai and Herman, 2009; Rinaman, 2011; Herman et al., 2016; Maniscalco et al., 2017; Chaves et al., 2021; Lamotte et al., 2021).

The current review focuses on the rodent prefrontal cortex, including a specific population of cells in the infralimbic cortex (IL) that is necessary for cardiovascular and endocrine responses to chronic stress. We also discuss the importance of prelimbic prefrontal cortex (PL) and highlight insular cortex effects on cardiovascular activity. Additionally, we discuss how these cortical regions may target the hypothalamus to trans-synaptically regulate neuroendocrine and cardiovascular autonomic effectors. The specific hypothalamic nuclei examined include the lateral hypothalamus (LH), dorsal medial hypothalamus (DMH), and posterior hypothalamus (PH), all of which innervate the PVN and regulate cardiovascular reactivity.

## Cortical Stress Regulation

### Medial Prefrontal Cortex

The medial prefrontal cortex (mPFC) is important for translating stress appraisal into adaptive behavioral and physiological responses through descending multi-synaptic circuits that target the HPA axis and autonomic effectors of cardiovascular activity. The mPFC is divided into dorsal and ventral subdivisions which have contrasting roles in acute and chronic stress reactivity (Radley et al., 2006; Mcklveen et al., 2013). Dorsal mPFC, PL in rodents, inhibits heart rate reactivity to acute stress (Tavares et al., 2009). Additionally, PL disinhibition reduces HPA axis reactivity to acute psychological stressors (restraint), while enhancing responses to physiological stressors (hypoxia; Jones et al., 2011). Further, GR signaling in the PL is necessary to inhibit corticosterone responses to acute restraint but not chronic variable stress (Mcklveen et al., 2013). Although the circuitry underlying PL effects on the cardiovascular system has not been directly queried, the HPA axis regulatory effects are mediated by synaptic relays in the bed nuclei of the stria terminalis (Radley et al., 2009; Johnson et al., 2019). Ultimately, these data suggest that the PL limits cardiovascular and HPA axis responses to acute psychological stressors with little evidence for involvement in chronic stress integration.

Subregions of human ventral mPFC exhibit altered activity in mood disorders (Drevets et al., 1997, 2008) and have been targeted for deep brain stimulation in treatment-resistant depression (Mayberg et al., 2005). Further, growing evidence supports a role for rodent ventral mPFC (IL) in cardiovascular and HPA axis regulation during chronic stress. Anatomically, the IL is largely composed of pyramidal glutamate neurons with a smaller population of GABAergic interneurons that regulate local activity (McKlveen et al., 2015, 2019). Principal IL glutamate neurons have unique connectivity compared to other cortical regions and innervate stress-regulatory structures throughout the amygdala, hypothalamus, and brainstem (Vertes, 2004). Further, chronic stress exposure shifts IL excitatory/inhibitory balance toward increased inhibition (Gilabert-Juan et al., 2013; McKlveen et al., 2016; Ghosal et al., 2020).

Initial studies of IL effects on cardiovascular stress reactivity found that non-specific synaptic blockers attenuated heart rate and blood pressure responses to acute restraint and fear conditioning (Resstel et al., 2006; Tavares et al., 2009). In contrast, IL NMDA activation reduces cardiovascular responses to air-jet stress (Camargos et al., 2012). Additionally, IL lesions increase stress-induced PVN activation, particularly in pre-autonomic cells (Radley et al., 2006). Taken together, these studies identified the importance of the IL for physiological stress responses but yielded contrasting results on whether the region increases or decreases stress responding. The contradictory results may relate to a lack of cellular specificity for lesion, pharmacology, and synaptic blockade approaches. Subsequent studies employed viral-mediated gene transfer to specifically target glutamate release from IL pyramidal cells. Here, genetic knockdown of pre-synaptic vesicular glutamate packaging in IL neurons increased tachycardic and pressor responses to acute restraint and elevated homecage arterial and pulse pressures during chronic variable stress (Schaeuble et al., 2019). Furthermore, decreased glutamate release from IL neurons during chronic stress increased vascular endothelial dysfunction, as well as histological indictors of cardiac and vascular hypertrophy. In terms of neuroendocrine regulation, decreased output from IL glutamate neurons increased HPA axis responses to acute restraint and exacerbated the effects of chronic stress on basal and stress-induced glucocorticoid hypersecretion (Myers et al., 2017). Altogether, these studies indicate that decreased glutamate release from IL neurons interacts with chronic stress to increase stress responding and promote susceptibility to cardiovascular pathologies.

Recent experiments utilizing optogenetic approaches to stimulate IL glutamate neurons in male and female rats revealed that activation of male IL pyramidal neurons restrains tachycardic and pressor reactivity to novel environment stress, as well as corticosterone and glucose responses to restraint (Wallace et al., 2021). Intriguingly, IL stimulation prior to chronic variable stress protected against stress-induced inward ventricular hypertrophy, an indicator of elevated sympathetic tone and cardiac load. While the mechanisms underlying protection from subsequent stress exposure are unclear, prior IL stimulation decreases net cardiac sympathetic drive. These effects may be explained by the persistent dendritic plasticity induced by optogenetic stimulation of IL glutamate neurons (Fuchikami et al., 2015). Although investigations of sex differences in cortical stress regulation are limited (Wallace and Myers, 2021), results from female IL glutamate neuron stimulation differ from males. In fact, female IL stimulation increases heart rate reactivity to novel environment stress and glucose responses to restraint. Collectively, these data indicate that IL glutamate neurons regulate cardiovascular and HPA axis responses to chronic stress in a sex-dependent manner. To investigate how IL output circuitry mediates autonomic-endocrine integration, IL functional connectivity and pre-synaptic innervation of downstream structures were quantified throughout the male forebrain (Wood et al., 2019). These data highlight IL inputs to key stress-integrative nuclei including the LH and DMH; however, significantly greater connectivity is evident in the PH. To date, the stress-regulatory effects of IL projections to specific hypothalamic nuclei have not been determined.

### Insular Cortex

Human imaging studies reveal that insular cortex shifts cardiac autonomic activity, possibly leading to arrhythmias (Oppenheimer and Cechetto, 2016). Furthermore, both human and rodent studies include the insula in the central autonomic network (Saper, 1982; Shoemaker and Goswami, 2015). From rostral to caudal, insular cortex is divided into the anterior insula (AI), posterior insula (PI), and an overlapping intermediate insula. Additionally, dorsal to ventral differences in cytoarchitecture lead to disgranular, granular, and agranular subdivisions (Gogolla, 2017). Although the insula has widespread limbic and visceral connectivity, the anatomical size and complexity have limited research on insular stress regulation. However, numerous studies have investigated the effects of insular stimulation on cardiovascular parameters, identifying effects of both AI and PI (Allen et al., 1991; Oppenheimer et al., 1991; Yasui et al., 1991). Specifically, electrical stimulation elicits tachycardia and modest arterial pressure increases in anesthetized rats (Ruggiero et al., 1987). Further experiments stimulating multiple insular regions to pinpoint the origin of cardiac regulation found two distinct regions of the rostral PI produce tachycardia and bradycardia (Oppenheimer and Cechetto, 1990). Systemic muscarinic and adrenergic antagonists indicate that insular chronotropic effects are mediated by either elevated (tachycardia) or decreased (bradycardia) sympathetic activity. This interpretation is further supported by recent findings that activation of rostral PI NMDA receptors inhibits brainstem pre-sympathetic regions causing bradycardia (Marins et al., 2016).

The results of functional studies align with monosynaptic anterograde tracing that indicates tachycardia-generating portions of the insula send efferents to pre-sympathetic regions including the LH, nucleus of the solitary tract, and parabrachial nucleus (Yasui et al., 1991). Efferents from bradycardic insular cortex have similar projection targets; although, connectivity with the pre-sympathetic regions is less dense. While anterograde tracing indicates that the LH is the primary hypothalamic target of insular cortex, retrograde tracing has identified projections to the DMH and PH (Abrahamson and Moore, 2001; Çavdar et al., 2001; Marins et al., 2020, 2021). In fact, recent reports specify that hemorrhagic stroke in the insula leads to disrupted cardiac sympathetic control and suggest that the DMH may be a downstream mediator (Marins et al., 2020, 2021). Altogether, decades of research have demonstrated that insular cortex influences cardiac autonomic activity, but the regional differentiation of the insula for stress integration remains unclear. Ultimately, more work is needed to understand the impact of insular subregions on stress adaptation and cardiovascular health.

While studies of insular cortex modulating cardiovascular or endocrine function during chronic stress have not been reported, recent work indicates chronic variable stress decreases expression of FosB/ΔFosB, a marker of long-term neural activity, throughout AI and PI (Pace et al., 2020). Interestingly, the long-term decrease in insular activity is dependent on the IL as knockdown of glutamate output from the IL prevents the effect. While insular FosB/ΔFosB-positive cells are glutamatergic, IL pre-synaptic terminals target both glutamatergic and GABAergic neurons in the AI and PI. These findings suggest that IL-insula communication during chronic stress modulates long-term excitatory/inhibitory balance of the insular cortices, which may have significant implications for visceral regulation.

## Effector Regions of the Hypothalamus

### Paraventricular Hypothalamus

**Figure 1:**
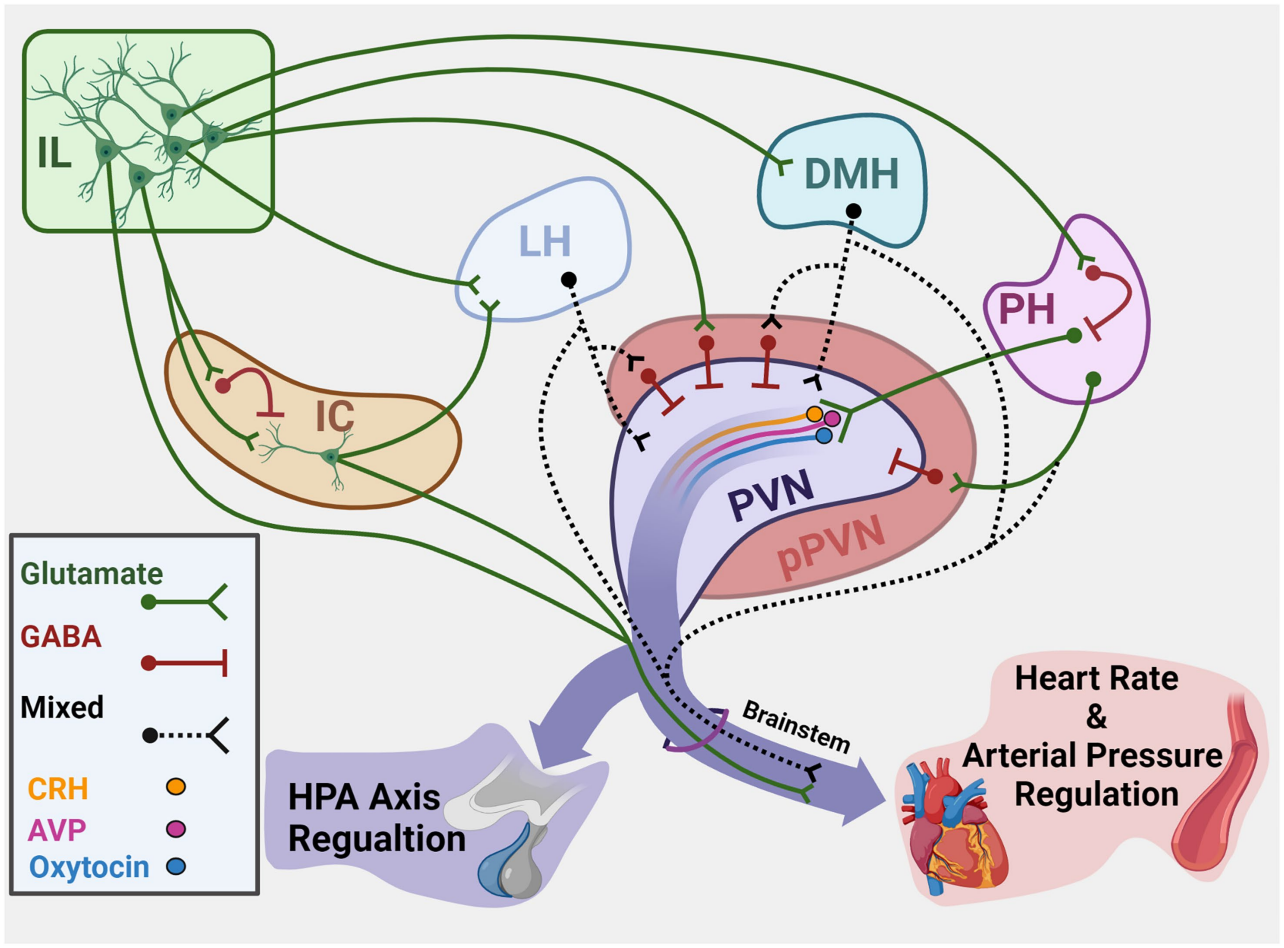
Summary of cortical–hypothalamic circuits mediating stress integration. Illustration of chronic stress-responsive cortical regions and hypothalamic targets that innervate the PVN and/or brainstem to regulate neuroendocrine and cardiovascular activity. Green represents glutamatergic neurons. Red represents GABAergic neurons. Black dashes represent mixed GABA and glutamate or neurochemically undefined anatomical connections. IL, Infralimbic cortex; IC, Insular cortex; LH, Lateral hypothalamus; DMH, Dorsomedial hypothalamus; PH, Posterior hypothalamus; PVN, Paraventricular nucleus of the hypothalamus; pPVN, Peri-paraventricular hypothalamus; CRH, Corticotropin-releasing hormone; AVP, Arginine vasopressin. Created with BioRender.com.

The PVN integrates hypothalamic and brainstem stress information to regulate both neuroendocrine and autonomic activity (Swanson and Kuypers, 1980). The region houses a diverse population of neurons that synthesize peptides implicated in stress reactivity including CRH, arginine vasopressin, and oxytocin. While PVN CRH release activates the HPA axis, multiple cell types give rise to brainstem and spinal projections to regulate sympathetic and parasympathetic balance (Ulrich-Lai and Herman, 2009). Notably, the descending cortical and limbic circuits that coordinate HPA axis and autonomic activity based on environmental context have limited direct innervation of the PVN (Roland and Sawchenko, 1993; Ulrich-Lai et al., 2011). However, numerous corticolimbic projections terminate in the GABAergic periphery of the PVN (peri-PVN) that surrounds neurosecretory cells (Cullinan et al., 2008; Ulrich-Lai and Herman, 2009; Sunstrum and Inoue, 2019). Though direct assessment of stress regulation by specific inputs to peri-PVN has been difficult, anterograde and retrograde tracing studies have identified hypothalamic regions that provide direct input to the PVN (Figure 1). Specifically, glutamatergic projections arise from the LH, DMH, and PH, among others (Ulrich-Lai et al., 2011). These three hypothalamic nuclei are well-positioned based on connectivity to integrate descending limbic information (Ledoux et al., 1988; Myers et al., 2014a). Furthermore, a portion of PVN-projecting neurons in the DMH and LH are GABAergic (Roland and Sawchenko, 1993), suggesting the nuclei may have both excitatory and inhibitory control over PVN neurons. Thus, cortical influences on HPA axis and cardiovascular reactivity are likely mediated trans-synaptically through innervation of hypothalamic regions that directly synapse in the PVN.

### Lateral Hypothalamus

The prefrontal and insular cortices, among other limbic regions, target the LH (Yasui et al., 1991; Vertes, 2004). Moreover, the LH provides direct glutamate and GABA input to the PVN (Roland and Sawchenko, 1993; Ulrich-Lai et al., 2011) and innervates pre-ganglionic autonomic neurons in the parasympathetic dorsal motor nucleus of the vagus and sympathetic intermediolateral column (Saper et al., 1976; Sun and Guyenet, 1986; Hahn and Swanson, 2010). Additionally, acute swim and restraint stressors activate cells in the LH (Cullinan et al., 1995); however, the phenotype of LH stress-responsive neurons has not been reported. The diversity of neurotransmitter and peptide messengers across the broad rostral to caudal breadth of the LH suggests a complex role in stress integration (Ziegler et al., 2002; Swanson et al., 2005; Ulrich-Lai and Herman, 2009). For instance, LeDoux and colleagues found that LH lesions reduce pressor responses to conditioned fear (Ledoux et al., 1988). In contrast, NMDA signaling in the LH inhibits cardiovascular responses to restraint stress through parasympathetic activation (Deolindo et al., 2013). Moreover, recent studies found that LH GABA_A_ and CRH receptor-1 antagonism decrease tachycardic responses to acute restraint stress (Gomes-de-Souza et al., 2019; Barretto-de-Souza et al., 2021). Together, these results suggest the LH may both increase and decrease cardiovascular reactivity. Despite direct PVN innervation, there are no reports to our knowledge of HPA axis modulation by the LH. However, it is worth noting that the LH more heavily targets the posterior PVN than the CRH-rich anterior PVN (Ulrich-Lai et al., 2011). Nevertheless, further analysis of LH stress integration is likely to elucidate specific subregional effects on sympathetic, parasympathetic, and neuroendocrine regulation.

### Dorsomedial Hypothalamus

The DMH is another prominent limbic relay for stress regulation. The DMH receives glutamatergic input from cortical circuits as well as GABAergic innervation from subcortical regions such as the amygdala (Myers et al., 2014a). Furthermore, the DMH robustly expresses immediately early gene markers following acute swim and restraint stress (Cullinan et al., 1995). DMH efferents target the PVN as well as pre-ganglionic sympathetic neurons (Saper et al., 1976), implying the region integrates stress-related processes (Ter Horst and Luiten, 1986, 1987; Thompson et al., 1996). The DMH projections to the PVN are both GABAergic and glutamatergic (Roland and Sawchenko, 1993; Ulrich-Lai et al., 2011), indicating the potential for both excitatory or inhibitory control of autonomic and endocrine stress responses. However, seminal work by DiMicco and colleagues used pharmacological approaches to interrogate the functional role of DMH neurotransmission in acute stress responding (DiMicco et al., 2002). Specifically, a series of studies in anesthetized rats found that GABA_A_ receptor antagonism or activation of ionotropic glutamate receptors (NMDA, AMPA, or kainate) in the DMH increases heart rate and blood pressure (Soltis and DiMicco, 1991a,b, 1992). Similar approaches in conscious rats found that DMH activation increases ACTH release, as well as stress-induced Fos in the PVN (Bailey and Dimicco, 2001; Morin et al., 2001). Moreover, GABA_A_ agonists in the DMH reduce heart rate, blood pressure, and ACTH responses to stress (Stotz-Potter et al., 1996a,b). In all, this work suggests that tonic GABAergic inhibition of the DMH reduces acute cardiovascular and endocrine stress responding while glutamate-mediated DMH activation enhances stress reactivity at least partially through the PVN. Although, autonomic aspects of DMH modulation have been hypothesized to be mediated by brainstem circuits (Fontes et al., 2011). Interestingly, the DMH is activated by both repeated restraint and chronic variable stress (Flak et al., 2012), yet the stress-integrative role of the DMH under conditions of prolonged stress requires more investigation.

### Posterior Hypothalamus

Cortical and limbic circuits also converge on the PH (Abrahamson and Moore, 2001; Çavdar et al., 2001) and the PH receives stress-activated inputs from multiple forebrain regions including the IL and PL (Myers et al., 2016). Although the PH is predominately glutamatergic, a portion of IL inputs appose PH GABA neurons, possibly regulating PH inhibition (Myers et al., 2016). Additionally, the PH sends glutamatergic projections to the PVN (Ulrich-Lai et al., 2011) and innervates pre-ganglionic sympathetic neurons (Saper et al., 1976). Furthermore, stress-reactive neurons in the rostral PH innervate stress-activated cells in the medial parvicellular PVN and pre-autonomic raphe pallidus (Nyhuis et al., 2016). Ultimately, the stress-responsive upstream and downstream connectivity of the PH implies a prominent role in stress integration. Similar to the DMH, early studies by Dimicco and colleagues identified the PH as a regulator of tachycardic and pressor responses under anesthesia (DiMicco et al., 1986). Specifically, both GABA release and GABA synthesis in the PH inhibit cardiovascular sympathetic activity (DiMicco and Abshire, 1987). In awake rodents, GABA modulation does not alter hemodynamics under basal conditions (Lisa et al., 1989). However, GABA_A_ signaling reduces heart rate and blood pressure responses to acute stress. More recent work demonstrates that CRH-mediated excitation in the PH increases HR *via* pre-sympathetic neurons in the rostral ventrolateral and ventromedial medulla, without affecting vagal activity (Gao et al., 2016). Taken together, these data indicate that the PH is both necessary and sufficient for acute sympathetic cardiovascular stress responses.

Multiple lines of converging evidence also implicate the PH in HPA axis facilitation. GABA_A_ agonists in the PH decrease ACTH during restraint (Myers et al., 2016) as well as corticosterone responses to acute restraint and audiogenic stress (Nyhuis et al., 2016). Furthermore, GABA antagonist-mediated disinhibition of the PH increases PVN Fos and elevates ACTH and corticosterone responses to restraint (Myers et al., 2016). In addition to CRH neurons, PH projections also target vasopressin- and oxytocin-producing cells in the PVN (Myers et al., 2016), suggesting the potential for broad neuroendocrine regulation. The PH also exhibits histological indicators of long-term activation during chronic variable stress but not repeated restraint (Flak et al., 2012). Altogether, there is considerable evidence that PH excitatory/inhibitory balance is important for autonomic and neuroendocrine stress integration.

## Conclusion

The widespread impact of stress on health-related quality of life and CVD in particular makes understanding stress physiology a crucial issue. Here, we discussed the function and connectivity of cortical and hypothalamic brain regions that integrate cardiovascular and neuroendocrine responses to stressful stimuli (Figure 1). The aggregate literature reviewed illustrates the hierarchal organization of descending cortical circuits that modulate stress-effector neurons through intermediary subcortical neurons. Cell-type-specific approaches have contributed to our understanding of stress reactivity and have the potential to uncover the basis of excitatory/inhibitory balance as it relates to chronic stress-induced pathologies. Although, it is important to note that few studies have directly examined cortical or hypothalamic stress integration in the context of chronic stress. Currently, there are reports of IL regulation of the endocrine and cardiovascular consequences of chronic stress, as well as IL inhibition of the insula during chronic stress (Mcklveen et al., 2013; Myers et al., 2017; Schaeuble et al., 2019; Pace et al., 2020; Wallace et al., 2021). However, to the authors’ knowledge, reports of functional hypothalamic cardiovascular–HPA axis stress integration are limited to acute stress studies. While we are beginning to unravel the roles of these networks in specific stress responses, many questions remain regarding autonomic-endocrine stress integration. Chiefly, most of the literature reviewed came from experiments with male subjects. Biological sex is an important factor for stress-related disease incidence and outcomes, yet the role of sex in stress responses across development and reproductive stages is largely unexplored. The experiments reviewed that included both sexes found marked differences in neural regulation of endocrine and autonomic reactivity (Wallace et al., 2021). Moreover, there is increasing evidence for ovarian hormone regulation of cardiac and vascular outcomes after chronic stress (Brooks et al., 2018; Finnell et al., 2018). Therefore, future studies examining the actions of sex steroids on cortical–hypothalamic circuits are vital for understanding the health burden of stress.

## Author Contributions

DS and BM contributed to the conceptualization of the review. DS performed the initial literature search, wrote the first draft of the manuscript, and created the illustration in BioRender. BM contributed additional literature and revised the manuscript. All authors approved the submitted version.

## Funding

This work was supported by AHA Predoctoral Fellowship 827519 to DS and NIH R01 HL150559 to BM.

## Conflict of Interest

The authors declare that the research was conducted in the absence of any commercial or financial relationships that could be construed as a potential conflict of interest.

## Publisher’s Note

All claims expressed in this article are solely those of the authors and do not necessarily represent those of their affiliated organizations, or those of the publisher, the editors and the reviewers. Any product that may be evaluated in this article, or claim that may be made by its manufacturer, is not guaranteed or endorsed by the publisher.
